# Consequences from use of reminiscence - a randomised intervention study in ten Danish nursing homes

**DOI:** 10.1186/1471-2318-10-33

**Published:** 2010-06-06

**Authors:** Claire Gudex, Charlotte Horsted, Anders Møller Jensen, Marianne Kjer, Jan Sørensen

**Affiliations:** 1 CAST - Centre for Applied Health Services Research & Technology Assessment, University of Southern Denmark, J.B. Winsløws Vej 9B, 5000 Odense C, Denmark; 2 Research and Development Division, VIOLA - Centre for the Life and Care of Elders, VIA University College, Campus Holstebro, Gl. Struervej 1, 7500 Holstebro, Denmark; 3 Occupational Therapist and Dementia Coordinator, Rødovre Municipality, Rødovre Parkvej 150, 2610 Rødovre, Denmark

## Abstract

**Background:**

Reminiscence is the systematic use of memories and recollections to strengthen self-identity and self-worth. The study aim was to investigate the consequences for nursing home residents and staff of integrating reminiscence into daily nursing care.

**Methods:**

In this randomised study, ten nursing homes were matched into two groups on the basis of location, type and size. In the period August 2006 - August 2007, staff in the Intervention Group were trained and supported in the use of reminiscence, involving individual and group sessions with residents as well as reminiscence boxes, posters and exhibitions. At baseline and again 6 and 12 months after the intervention start, data were collected on residents' cognitive level, agitated behaviour, general functioning and proxy-assessed quality of life, as well as on staff well-being and job satisfaction. Mixed linear modelling was used to analyse differences in outcome between the intervention and control groups.

**Results:**

Project drop-out rates were 32% for residents and 38% for nursing staff. Most staff in the Intervention Group considered reminiscence a useful tool that improved their communication with residents, and that they would recommend to other nursing homes. There were no significant differences between residents in the Intervention and the Control Group in cognitive level, agitated behaviour or general functioning. Residents in the Intervention Group showed significant higher score at 6 months in quality of life subscale 'Response to surroundings', but there was no significant difference at 12 months.

Positive effects of reminiscence were observed for all staff outcome measures, the only exception being SF-12 self-rated physical health. At 6 months after start of reminiscence, staff in the Intervention Group had significantly better scores than those in the Control Group for Personal accomplishment, Emotional exhaustion, Depersonalisation, 'Attitude towards individual contact with residents' and SF-12 self-rated mental health. At 12 months after start of reminiscence, staff in the Intervention Group had significantly better scores than those in the Control Group for Emotional exhaustion and 'Professional role and development'.

**Conclusions:**

The use of reminiscence appeared to have little long-term effect on the nursing home residents. Nursing staff in the Intervention Group experienced greater satisfaction with professional roles and developed a more positive view of the residents.

**International Standard Randomised Controlled Trial Number Register: ISRCTN90253170**.

## Background

A major change in the Danish elderly sector over the last several years is the greater focus on providing services that support the maintenance of the elderly person's personal integrity and self-identify [1]. This is in part derived from psychologist Tom Kitwood's theories regarding person-centred care of people with dementia [2]. A central message here is that emotions and experiences should play a major role in the interaction between the person with dementia and the carer, and the uniqueness of each individual's experience must always be taken into account. An understanding of the behaviour and reactions of the person with dementia is crucial in ensuring that the most appropriate interaction will occur.

Reminiscence is an approach that nursing home staff can use to strengthen residents' feelings of self-identity, and an introduction to the method is now included in many training courses for nursing home staff in Denmark. Reminiscence can be described as '*the systematic use of memories and recollections to reawaken or strengthen self-identity and self-worth' *(translated from [3]). The approach has its basis in Robert Butler's work on 'life review' [4]. However, while life review is an intrapersonal approach undertaken only at individual level with an aim to resolve internal conflicts stemming from past experiences [5], reminiscence is an interpersonal approach undertaken either at individual level or group level with the aim of eliciting and sharing pleasant memories and experiences in an informal and conflict-free manner.

The rationale behind the use of reminiscence is thus an attempt to [6-9]:

• Strengthen feelings of the individual's identity and self-worth

• Re-establish feelings of coherence and control over one's own life

• Encourage the individual to (continue to) value his/her own life and to remember and re-experience happy events and associated positive feelings.

Reminiscence can be beneficial for elderly people with depressive symptoms. A meta-analysis [10] of 20 studies (of which 15 were RCTs) found that the effect size of reminiscence was equivalent to that of antidepressive medication and cognitive behavioural therapy.

Reminiscence is also expected to benefit elderly people with dementia. Although individuals with advanced dementia have often lost the ability to actively consider their past, their distant memory is often intact as is the ability to develop relationships and interact with their environment [11]. A Cochrane meta-analysis [12] of four randomised controlled studies found some evidence for improved cognitive function and fewer depressive symptoms among elderly people with dementia who had participated in reminiscence, but also noted that methodological deficiencies (such as small study samples and non-blind assessments) and the large variation in the reminiscence approaches used prevented robust conclusions. Other studies of the use of reminiscence among elderly with dementia have reported minor improvements in social functioning and less problem behaviour [7] and no difference in agitated behaviour (compared to a group undergoing stimulation therapy) [13]. In determining the impact of reminiscence, it is important to take account of individual differences among residents such as level and type of dementia, communication skills and environmental context [14].

Few studies have investigated the effect of reminiscence on nursing staff. In two studies where reminiscence was only one of several approaches implemented in the intervention group, small improvements were found in job satisfaction and stress levels [15,16]. Reminiscence has also been reported to increase the nursing staff's knowledge of the residents' life history, leading to the staff finding it easier and more enjoyable to talk with the residents about personal topics [17,18].

Given the lack of a standard approach to reminiscence in the elderly sector, and limited evidence from large randomised studies, the aim of the current study was to use an RCT approach to investigate the consequences for nursing home residents and staff of integrating reminiscence into daily nursing care, i.e.

• Consequences for residents in terms of quality of life, agitated behaviour and cognitive and physical function

• Consequences for staff in terms of job satisfaction, stress/burnout and health status.

The specific study questions were whether use of reminiscence could:

i) Reduce (and perhaps reverse) the deterioration that one could expect to see over time in the functional level of nursing home residents, and especially those with low cognitive function

ii) Improve the staff's assessment of their satisfaction with nursing care and their work situation, work-related burnout and own (mental) health.

The reminiscence approach used in the current study builds further on the approach used by the Danish Centre for Reminiscence (Nørrebro Erindringscenter). Greater emphasis has been given to categorising reminiscence as general, specific or spontaneous, however, in order to illustrate to the nursing staff that reminiscence can be used in a wide range of situations, also without pre-planned sessions or tours out of the home.

## Methods

The study was undertaken as a randomised, matched intervention study. Ten nursing homes were matched by the project team into two groups on the basis of location (municipality, urban/rural), type (traditional institutional multi-storey building with long corridors vs. home-like units with central communal rooms) and size (under and over 45 residents). The two lists of five nursing homes were then placed in two blank sealed envelopes; a colleague external to the project group was asked to arbitrarily choose one envelope; the five nursing homes named in this envelope became the Intervention Group (IG), who implemented reminiscence. The remaining five nursing homes became the Control Group (CG), who continued with usual nursing care. Nursing homes were not told of their group until after the second baseline data collection was completed. Blinding of nursing staff with respect to intervention was not possible, although the project interviewers were not formally informed of which group the nursing homes were in.

Because the variation in resident characteristics between nursing homes was unknown, sample size calculations were made at individual level i.e. according to resident characteristics rather than nursing home characteristics. These indicated that approximately 350 residents would be required to enter the study in order to achieve statistical significant results. This was based on the following assumptions:

• That a reduction in residents' agitated behaviour, as measured by the Cohen-Mansfield Agitation Inventory (CMAI), was the primary outcome

• That a change of 4.15 in CMAI score represented a clinically relevant effect [19]

• A CMAI score standard deviation of 16.8 [20]

• That 30% of residents would drop out over the course of the study due to worsening health and death

• A significance level of 5% and power of 80%.

### Recruitment of participants

A previous study [21] had shown that *nursing homes *in the region had an average size of 35 residents; the aim was thus to recruit ten nursing homes that did not already use reminiscence on a systematic basis. In August 2005 letters of invitation were sent to 35 municipalities in the Mid-Jutland Region where there was at least one nursing home with a minimum 35 residents; these letters were followed up by personal visits to explain the nature and aim of the project. Twenty-six municipalities declined to participate and a further two were excluded due to extensive building projects and/or staff training in the area. From the remaining seven municipalities there were 14 nursing homes that were willing to be randomised; four of these were excluded after closer examination (two nursing homes already used reminiscence in daily care, one was under new management and the fourth had recently undergone extensive staff training). The ten participating nursing homes were all publicly owned and at recruitment (February 2006) had a total of 447 residents. Nursing home managers were requested not to undertake any major organisational or staff-related changes over the course of the project.

All *residents *who lived permanently in the nursing home were included in the study, with the following exceptions: i) residents with advanced dementia living in protected-environment units within the nursing homes; these were typically small units with specialised nursing staff who already used reminiscence in daily care, ii) residents who were bed-ridden or terminally ill (8), were on short-term placement or waiting to move to a protected-environment unit (5), or were known to become aggressive in new situations (2). The nursing staff informed residents individually both orally and in writing about the study and asked for signed consent; all relatives/visitors were also informed of the study and were asked for consent in cases where the resident was unable to give it.

All permanent *nursing staff *involved in the daily personal care of the residents were included in the study, i.e. nurses, nurse aids and occupational therapists. Administrative and service staff were excluded. All the nursing staff in the Intervention Group were expected to participate in reminiscence training and to implement the method in their daily work.

### Intervention

Nursing home staff in the Intervention Group attended a standardised course in reminiscence (two whole and two half-days over a 9-month period) that comprised formal teaching, group work and discussion sessions (conducted by MK). During the intervention period (August 2006 - August 2007) nursing staff were expected to use three forms of reminiscence:

• General reminiscence: group sessions run by 1-2 nurses for typically 2-8 residents with similar backgrounds or interests; structured around a chosen theme, often based on a reminiscence box containing a variety of tools, photos, books, music etc. and involving senses of sight, touch, smell, hearing.

• Specific reminiscence: sessions for 1-2 residents structured around a theme and tailored to the individual resident's communication needs; often using family photos, reminiscence games and activities e.g. baking.

• Spontaneous reminiscence: informal use of comments during regular daily activities (e.g. dressing, meal-times, preparing for bed) to elicit a resident's memories of earlier life experiences; can start as an individual contact that spreads to involve others sitting nearby.

Immediately after each general and specific reminiscence session, the participating staff assessed each resident's level of engagement according to a 5-point scale.

**Figure 1:**
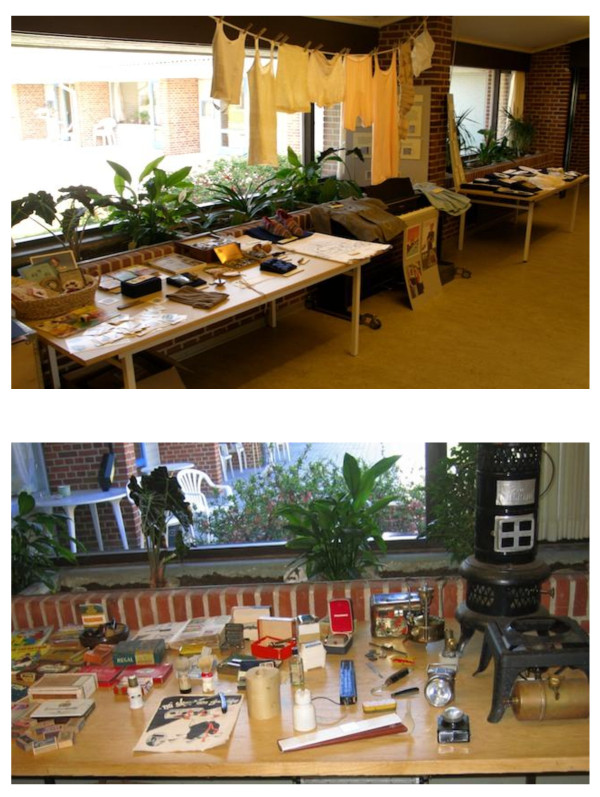
**Examples of reminiscence exhibitions**. *The ladies' exhibition 'Darning and embroidery' *included a home-sewn and darned child's coat, button box, embroidery patterns, crocheted collars, lace handkerchiefs, sewing box, washing line and pegs. *The gentlemen's exhibition *included shaving equipment, old electrical plugs, rationing stamps, technical books, primus stove, a peat cutter and a knife-grinder's cycle.

Reminiscence themes used included childhood, kitchen and housework, rural life, family life, occupations, leisure activities and traditions; sessions could also take the form of games and tours to local historical buildings and museums. Thirty reminiscence boxes were circulated between the five IG nursing homes during the intervention period. These boxes could also be used to make small exhibitions, which could be visited by staff, residents and visitors (Figure 1). Posters showing historical objects were also made available to each nursing home.

Separate meetings were held (by MK and AM) with each nursing home's management and selected project contact persons in order to ensure support and a coordinated effort for reminiscence activities. A reminiscence trainer (AM) visited each nursing home 5-8 times during the intervention period to provide guidance and encouragement in the use of reminiscence material and activities.

The importance of documenting the individual resident's life history was emphasised to both staff and relatives, with examples to illustrate how knowledge of previous life experiences could be used to make contact with residents, to determine meaningful activities for the resident and to help residents express their thoughts and feelings.

### Data collection

Baseline data were collected among residents in March, May and August 2006. Data collection was repeated 6 and 12 months after start of reminiscence training, i.e. March and August 2007. Selection of instruments was based on relevance for an institutionalised population, wide use with a validated version available in Danish, and practical ease of completion. The instruments were tested in a pilot study prior to the main study.

At the first baseline data collection, nursing staff completed the questionnaires on residents' agitated behaviour, quality of life and general level of functioning together with a project interviewer. Hereafter the nursing staff completed the questionnaires themselves. Project interviewers assessed resident's cognitive level. The six interviewers were nurses and/or community dementia coordinators; they attended a training day where they were instructed in the use of the questionnaires and cognitive tests.

Nursing staff completed a questionnaire about work-related burnout, job satisfaction and own health four times in the course of the study, i.e. March and August 2006 (baseline) and March and August 2007 (6 months and 12 months after start of reminiscence training, respectively).

### Outcome measures

**CMAI **(Cohen-Mansfield Agitation Inventory) assesses the frequency of 29 types of agitated behaviour within the previous two weeks, as well as how disturbing these behaviours are for other residents and staff [22]. Frequency of each behaviour is scored from 0 (Never) to 6 (Several times an hour); responses for the individual behaviours are summed to give a total Frequency score between 0 and 174 [23]. Four subscale scores can be calculated for physically aggressive and non-aggressive behaviour and verbally aggressive and non-aggressive behaviour, respectively. Disturbance is scored from 0 (Not at all disturbing) to 4 (Extremely disturbing); responses for the individual behaviours are summed to give a total Disturbance score between 0 and 116 [24]. A higher score reflects more frequent or disturbing agitated behaviour.

**ADRQL **(Alzheimer Disease Related Quality of Life) was developed to assess the quality of life of people with Alzheimer's disease, but is also used with other forms of dementia. It comprises 47 items categorised under five domains: social interaction, awareness of self, response to surroundings, enjoyment of activities and feelings/mood [25]. The Yes/No responses to each item are weighted; both the total score and the five subscale scores are expressed as a percentage of the total possible score and range between 0 and 100. A higher score reflects better quality of life for the individual resident (as perceived by the nursing staff). As the ADQRL was not available in Danish, it was translated by the project group using forward and backward translation followed by testing in the pilot study [26].

**GBS **(Gottfries-Bråne-Steen scale) assesses the general functioning of people with dementia and comprises 27 items categorised under intellectual, emotional and ADL (activity of daily living) impairments, as well as symptoms of dementia [27]. Responses (0 to 6) to the items are summed to subscale scores and to an overall score that ranges between 0 and 162. A higher score reflects a lower level of functioning.

**MMSE **(Mini-Mental State Examination) measures cognitive function, including orientation in time and place, recall, language, attention and calculation. The score for each item is summed to a total score that ranges between 0 and 30. A higher score reflects better cognitive function; a score of 24-30 is considered normal, score 18-23 reflects mild cognitive deterioration and score under 18 reflects severe cognitive deterioration [28].

**SIB-S **(Severe Impairment Battery - Short Form) was used with residents who had a MMSE score less than 15, as MMSE is a less sensitive measure for people with severe dementia. The original SIB instrument has 51 items [29]; the same authors have developed the short version, that has 25 items with a total score ranging between 0 and 50 [30]. A higher score reflects better cognitive function (though still in the lowest levels of cognitive functioning).

**MBI-HSS **(Maslach Burnout Inventory - Human Services Survey) assesses burnout among people who work professionally with others, including those in the health services [31]. The 22 items (each with six response categories ranging from 'never' to 'every day') are categorised into three subscales: personal accomplishment (scale from 0 to 48, where a high score reflects feelings of competence and successful work achievement), emotional exhaustion (scale from 0 to 54, where a high score reflects feelings of being emotionally overextended and exhausted by one's work) and depersonalisation (scale from 0 to 30, where a high score reflects an insensitive and impersonal response towards recipients of one's care).

**SNCW **(Satisfaction with Nursing Care and Work Assessment) scale comprises 34 items (each with five response categories ranging from 'strongly agree' to 'strongly disagree') relating to work environment and personal development [32,33]. Only 11 items were used in the current study, as the others were covered by the other instruments. The original Swedish instrument was translated to Danish using forward translation followed by testing in the pilot study. Factor analysis was then used to categorise the 11 SNCW items into dimensions; four dimensions were identified: Professional role and development, Work environment and staff collaboration, Residents' level of care and Individual contact with residents. The lower the score, the greater the level of satisfaction with nursing care and work or the more positive the staff attitude.

**SF-12v2 **(Short Form-12 version 2) is a generic measure of health status comprising 12 items; scores can be calculated for self-assessed physical health (PCS) and mental health (MCS), where the average score for the general population is set at 50 [34,35]. A score over 50 implies that own health is assessed as better than average. The question on general health perception asks 'In general, would you say your health is excellent, very good, good, fair or poor?'

Staff experiences with the actual implementation of reminiscence were further investigated in two ways: i) through additional items in the staff questionnaire, that only the IG group answered, and ii) through personal interviews with 14 selected staff members in the IG group, conducted by CH and CG at the end of the study. A semi-structured interview guide was used to enquire about staff attitudes towards the reminiscence activities and their impact on staff and residents, the level of implementation of reminiscence and the prerequisites for a successful implementation. The informants, who were selected by the manager of each nursing home, came from different units within the home and included staff with both positive and more negative attitudes towards the implementation of reminiscence.

### Statistical analysis

There were few missing data (under 4%) on instrument items. Missing data on ADRQL items were adjusted for as recommended by the instrument's developers. Missing data on other instrument items meant that total scores could not be calculated; data for these respondents were thus excluded from the analysis of instrument scores.

Differences between IG and CG at baseline were assessed using Chi-squared test or Fisher's exact test for categorical variables, t-tests for continuous normally distributed data and Mann-Whitney test for continuous non-normally distributed data.

Mixed linear modelling was used to analyse differences in outcome between the intervention and control groups while taking account of intra-individual correlations; this approach is fairly robust to missing observations, which are assumed to be missing at random [36-38]. The outcome scores were treated as continuous dependent variables. The covariates used in the main model were: randomisation to intervention group (iv = 1), time of observation (baseline BL1, BL2 and BL3 (reference) and T6 and T12 i.e. 6 and 12 months after start of reminiscence) and interaction variables T6*iv and T12*iv. The estimated models allowed each participant to deviate from the overall mean score (random intercept model). Early analysis identified significant differences in score development for residents with normal cognitive functioning (MMSE > 23) in comparison to residents with cognitive impairment; an indicator variable for cognitive function (normal at baseline 3 = 1) was thus also included as a covariate in the outcome analysis for residents. Further models were estimated to allow both intercept and slope variations for the interaction variables; these did not improve the model fit, however, and are not reported here. The linear mixed modelling was carried out using STATA version 11. A 5% level of significance was used.

## Results

### Characteristics of nursing home residents

**Figure 2:**
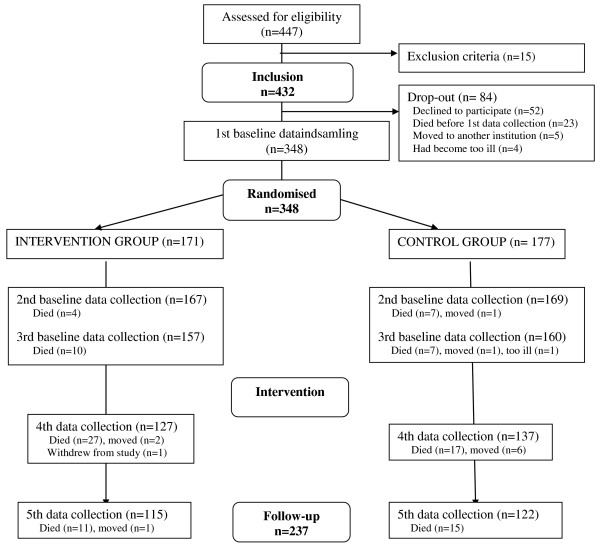
**Flow chart of nursing home residents' participation in the reminiscence study**.

In total, 52 residents (or the family on behalf of the resident) declined to participate in the study and a further 32 dropped out before the baseline data collection, leaving 348 residents (Figure 2). The drop-out rate over the project period was 32% for residents. Of the 111 residents who failed to complete the study, 98 had died, 11 had moved out of the nursing home, 1 withdrew study participation and 1 had become too ill. There were no significant differences in sociodemographic characteristics between the 348 residents who started the study and the 237 residents who completed the study (Table 1). Drop-out rates were similar in IG and CG.

**Table 1:** Characteristics of all participating residents and those who completed the study.

	*Baseline* *(n = 348)*	*Study completion* *(n = 237)*
Women	68%	68%
Mean age (s.d.)	82.3 yrs (9.5)	81.9 yrs (9.8)
Mean (s.d.) length of residence	2.1 yrs (3.1)	2.3 yrs (3.3)
On dementia medication within the past year	8%	6%
Has dementia diagnosis	19%	18%
Reduced physical mobility (incl. wheelchair use)	77%	73%
Treated for depression within past 1 year	47%	45%

There were no significant differences between residents in IG and CG at baseline with respect to sociodemographic variables or scores for agitated behaviour, quality of life, general functioning or cognitive function. According to the baseline MMSE score, approximately half (47%) of the residents had severe cognitive impairment (score < 18) and 24% had normal cognition (score > 23).

### Effect of reminiscence on residents

Tables 2 and 3 present the estimated parameters and confidence intervals from the mixed modelling analysis of outcomes for residents.

**Table 2:** Results of mixed modelling analysis of outcome scores for residents in the Intervention and Control Groups.

	*Frequency of agitated behaviour (CMAI)*	*Disturbance from agitated behaviour (CMAI)*	*Quality of life (ADRQL)*	*General functioning (GBS)*	*Cognitive function (MMSE)*	*Cognitive function (SIB-S)*
BL1	**1.7 (0.7;2.7)**	0.8 (-0,3;1.8)	0.2 (-0.9;1.4)	**-4.7 (-6.7;-2.7)**	-0.4 (-0.8;0.1)	0.2 (-1.8;2.1)
BL2	-0.2 (-1.1;0,8)	-0.2 (-1,3;0.9)	0.2 (-0.9;1.4)	**-2.6 (-4.6;-0.6)**	Not measured	Not measured
BL3	Reference	Reference	Reference	Reference	Reference	Reference
T6	1,0 (-0,4;2,4)	0.9 (-0.6;2.5)	-0.3 (-1.9;1.3)	2.6 (-0.2;5.4)	Not measured	**-3.8 (-6.5;-1.2)**
T12	**1.5 (0.1;3.0)**	0.3 (-1.3;1.8)	**-2.1 (-3.7;-0.5)**	**5.2 (2.3;8.1)**	-0.6 (-1.3;0.1)	**-3.8 (-6.6;-0.9)**
iv	1.2 (-1.1;3.4)	-0.0 (-1.6;1.6)	-1.4 (-4.0;1.1)	-1.0 (-6.7;4.7)	-0.1 (-1.3;1.1)	-1.7 (-6.5;3.1)
T6*iv	0.1 (-1.8;1.9)	-1.0 (-2.9;1.0)	0.7 (-1.4;2.8)	-2.0 (-5.6;1.6)	Not measured	3.2 (-0.4;6.9)
T12*iv	-1.5 (-3.4;0.4)	-0.2 (-2.3;1.8)	0.6 (-1.5;2.7)	2.7 (-1.1;6.4)	0.6 (-0.3;1.5)	-2.6 (-6.6;1.3)
MMSE > 23	**-7.6 (-10.2;-5.1)**	**-3.7 (-5.6;-1.8)**	**12.0 (9.0;14.9)**	**-38.6 (-45.2;-31.9)**	**10.8 (9.5;12.2)**	Not applicable
Constant	**10.4 (8.6;12.2)**	**6.6 (5.2;8.0)**	**78.8 (76.7;80.8)**	**59.4 (55.0;63.9)**	**15.4 (14.4;16.3)**	**34.7 (31.2;38.1)**

n(obs/residents)	1391/342	987/304	1476/344	1393/348	696/331	343/121
Log-restricted likelihood	-4902.8	-3290.9	-5429.7	-5963.5	-1972.2	-1259.7

Figures are modelled parameter estimates (95% confidence intervals).

BL1 = indicator for baseline 1; BL2 = indicator for baseline 2; BL3 = indicator for baseline 3; T6 = indicator for t = 6 months after start of intervention; T12 = indicator for t = 12 months after start of intervention; iv = indicator for intervention group; T6iv = interaction between T6 and iv; T12iv = interaction between T12 and iv; MMSE > 23 = indicator for baseline cognitive function in normal range.

**Table 3:** Results of mixed modelling analysis of five ADRQL subscores for residents in the Intervention and Control Groups.

	*Social interaction (SI)*	*Awareness of self (AS)*	*Response to surroundings (RS)*	*Enjoyment of activities (EA)*	*Feelings/mood (FM)*
BL1	0.2 (-1.5;2.0)	**3.5 (1.5;5.5)**	-0.1 (-1.7;1.4)	1.9 (-1.2;4.9)	**-1.8 (-3.2;-0.3)**
BL2	0.7 (-1.0;2,5)	-0.4 (-2.5;1.6)	0.9 (-0.7;2.5)	0.5 (-2.6;3.5)	-0.0 (-1.4;1.4)
BL3	Reference	Reference	Reference	Reference	Reference
T6	0.7 (-1.7;3.2)	-0.1 (-3.0;2.7)	-0.3 (-2.5;1.9)	-0.7 (-5.0;3.6)	-0.6 (-2.7;1.4)
T12	-0.6 (-3.1;1.8)	**-3.5 (-6.4;-0.6)**	-0.1 (-2.4;2.1)	-3.1 (-7.5;1.3)	**-2.6 (-4.6;-0.5)**
iv	-1.0 (-4.2;2.2)	0.1 (-3.6;3.8)	-2.7 (-5.5;0.0)	-2.9 (-7.8;2.0)	-1.6 (-4.4;1.3)
T6*iv	-0.7 (-3.8;2.4)	0.2 (-3.5;3.9)	**3.4 (0.6;6.2)**	-1.0 (-6.5;4.6)	1.2 (-1.4;3.8)
T12*iv	-1.5 (-4.8;1.8)	1.3 (-2.6;5.1)	0.5 (-2.4;3.5)	2.8 (-3.0;8.5)	1.1 (-1.6;3.8)
MMSE > 23	**10.7 (7.1;14.4)**	**18.2 (14.0;22.4)**	**8.7 (5.5;11.8)**	**20.6 (15.1;26.1)**	**8.1 (4.8;11.4)**
Constant	**76.8 (74.2;79.5)**	**68.3 (65.2;71.4)**	**88.5 (86.2;90.8)**	**64.5 (60.4;68.4)**	**86.2 (83.9;88.5)**

n(obs/residents)	1476/344	1476/344	1473/344	1474/344	1476/344
Log-restricted likelihood	-5979.0	-6211.2	-5798.2	-6749.01	-5732.4

Figures are modelled parameter estimates (95% confidence intervals).

BL1 = indicator for baseline 1; BL2 = indicator for baseline 2; BL3 = indicator for baseline 3; T6 = indicator for t = 6 months after start of intervention; T12 = indicator for t = 12 months after start of intervention; iv = indicator for intervention group; T6iv = interaction between T6 and iv; T12iv = interaction between T12 and iv; MMSE > 23 = indicator for baseline cognitive function in normal range.

#### a) Agitated behaviour (CMAI)

• Agitated behaviour was more frequent over time for both groups and was significantly more frequent at 12 months. Residents in IG had less frequent agitated behaviour at 12 months than CG, but this difference was not significant.

• Perceived disturbance from agitated behaviour showed no change over time, and there were no differences between IG and CG.

• Normal cognitive score at baseline (MMSE > 23) was significantly associated with less frequent agitated behaviour and less perceived disturbance from agitated behaviour.

#### b) Quality of life (ADRQL)

• Quality of life scores worsened over time for both groups and the reduction after 12 months was significant. There were no differences in score change over time between IG and CG.

• The subscale 'Response to surroundings' showed a significant higher score at 6 months for IG than for CG, but no significant difference between IG and CG at 12 months (Table 3).

• No significant differences between IG and CG were indentified on the other four subscales.

• Normal cognitive score at baseline (MMSE > 23) was significantly associated with higher quality of life score.

#### c) General functioning (GBS)

• GBS score showed deterioration over time in both groups and showed significant deterioration at 12 months. There were no significant differences between IG and CG.

• Normal cognitive score at baseline (MMSE > 23) was significantly associated with better general functioning.

#### d) Cognitive function (MMSE and SIB-S)

• There was deterioration in cognitive function over time in both groups and this was significant for SIB-S (and close to significance for MMSE). Regarding residents with *lower *baseline cognitive function (i.e. where SIB-S was used to assess cognitive function), those in IG showed a higher score at 6 months than residents in CG, although this difference was not significant.

There were no significant differences in score change on any instrument between residents who participated in many reminiscence sessions and residents who participated in fewer sessions, even after taking baseline cognitive function into account (data not shown).

According to the staff assessments, residents participating in the reminiscence sessions typically showed 'clear signs of interest, engagement or enjoyment' or 'varying interest, mostly positive'; very few residents became angry or irritable during the sessions. There was no consistent pattern in individual resident's level of engagement in reminiscence sessions over the study period. The association between level of engagement and score change was thus not explored.

### Characteristics of nursing home staff

**Figure 3:**
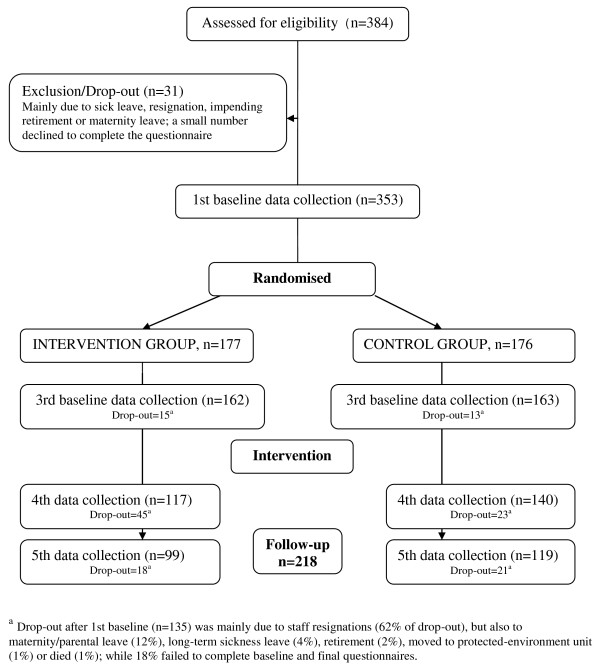
**Flow chart of nursing home staff participation in the reminiscence study**.

The drop-out rate over the project period was 38% for nursing staff. Of the 135 staff who failed to complete the study, 111 were no longer working at the nursing home and 24 did not complete follow-up questionnaires (Figure 3). There were no significant differences in either sociodemographic characteristics or instrument scores (SF-12 and MBI-HSS) between the 353 nursing staff who participated at baseline and the 218 staff who completed the study. Drop-out rates were similar in IG and CG (overall mean 38.2%), but there was large variation across nursing homes, ranging between 14% and 62%.

The average age of the nursing staff (of whom 97% were women) was 47 years and the average length of employment in the nursing home 8 years. Most (87%) were nurse aids (1.5 years' training) or nurse assistants (3 years' training); the remainder were nurses (3.5 years' training) and therapists. Staff in CG had been employed longer than those in IG (10.1 vs. 6.1 years, p < 0.001) - presumably because more of the homes in IG were relatively new; otherwise there were no significant differences between the staff in IG and CG.

At baseline approximately half of the nursing staff respondents rated their own health as 'excellent' or 'very good'; under 10% rated it as 'poor' or 'fair'; there were no significant score differences between staff in IG and CG. According to the baseline MBI-HSS scores, most of the staff respondents experienced high personal accomplishment (77%), low emotional exhaustion (75%) and low depersonalisation (91%). Staff in IG reported significantly higher baseline emotional exhaustion than staff in CG (mean score IG = 13.0 vs. CG = 10.4; p = 0.035), as well as a significantly worse score on the SNCW-derived domain of Work environment & staff collaboration (IG = 2.7 vs. CG = 2.1, p = 0.004).

### Effect of reminiscence on staff

Tables 4 and 5 present the estimated parameters and confidence intervals from the mixed modelling analysis of outcomes for staff.

**Table 4:** Results of mixed modelling analysis of MBI and SF-12v2 scores for staff in the Intervention and Control Groups.

	*MBI - PA (Personal accomplishment)*	*MBI - EE (Emotional exhaustion)*	*MBI - DPM* *(Depersonalis- ation)*	*SF-12v2 PCS (Physical component)*	*SF-12v2 MCS (Mental component)*
BL1	-1.0 (-0.7;0.5)	-0.8 (-1.6;0.1)	-0.2 (-0.5;0.1)	**0.9 (0.1;1.7)**	-0.9 (-1.9;0.0)
BL3	Reference	Reference	Reference	Reference	Reference
T6	-0.5 (-1.3;0.4)	**1.5 (0.3;2.7)**	0.1 (-0.4;0.6)	0.3 (-0.9;1.4)	**-1.9 (-3.3;-0.6)**
T12	-0.5 (-1.3;0.4)	1.1 (-0.1;2.4)	0.1 (-0.4;0.6)	-0.6 (-1.7;0.6)	-0.7 (-2.1;0.7)
iv	-0.6 (-1.7;0.5)	**2.1 (0.4;3.9)**	**0.7 (0.0;1.3)**	0.7 (-0.8;2.3)	**-1.6 (-3.2;-0.0)**
T6*iv	**1.5 (0.4;2.7)**	**-2.3 (-4.0;-0.6)**	**-0.8 (-1.4;-0.1)**	-1.3 (-2.9;0.2)	**1.9 (0.0;3.8)**
T12*iv	0.0 (-1.2;1.2)	**-2.1 (-3.8;-0.3)**	-0.3 (-1.0;0.4)	-0.0 (-1.7;1.6)	1.3 (-0.7;3.3)
Constant	**42.4 (41.5;43.2)**	**11.5 (10.2;12.8)**	**2.1 (1.6;2.5)**	**50.9 (49.7;52.1)**	**53.6 (52.4;54.9)**

n(obs/residents)	999/344	999/344	999/344	999/344	999/344
Log-restricted likelihood	-2947.7	-3361.3	-2405.4	-3270.9	-3390.1

Figures are modelled parameter estimates (95% confidence intervals).

BL1 = indicator for baseline 1; BL2 = indicator for baseline 2; BL3 = indicator for baseline 3; T6 = indicator for t = 6 months after start of intervention; T12 = indicator for t = 12 months after start of intervention; iv = indicator for intervention group; T6iv = interaction between T6 and iv; T12iv = interaction between T12 and iv.

**Table 5:** Results of mixed modelling analysis of four SNCW factors for residents in the Intervention and Control Groups.

	*Professional role and development*	*Work environment & staff collaboration*	*Attitude towards residents' level of care*	*Attitude towards contact with residents*
BL1	-0.0 (-0.2;0.2)	0.1 (-0.1;0.2)	-0.0 (-0.2;0.1)	0.1 (-0.1;0.2)
BL3	Reference	Reference	Reference	Reference
T6	0.2 (-0.1;0.6)	-0.0 (-0.3;0.2)	**-0.2 (-0.4;-0.0)**	0.1 (-0.2;0.3)
T12	**0.5 (0.2;0.9)**	0.0 (-0.2;0.3)	-0.0 (-0.2;0.2)	-0.0 (-0.2;0.2)
iv	0.3 (-0.1;0.8)	**0.5 (0.2;0.8)**	**0.2 (0.0;0.4)**	0.2 (-0.1;0.5)
T6*iv	-0.0 (-0.5;0.4)	0.0 (-0.3;0.4)	-0.0 (-0.3;0.2)	**-0.4 (-0.7;-0.1)**
T12*iv	**-0.5 (-1.0;-0.1)**	-0.3 (-0.7;0.0)	-0.2 (-0.5;0.0)	-0.2 (-0.6;0.1)
Constant	**3.1 (2.8;3.5)**	**2.1 (1.9;2.3)**	**1.5 (1.4;1.7)**	**2.6 (2.3;2.8)**
n(obs/residents)	1076/350	1076/350	1076/350	1076/350
Log-restricted likelihood	-2215.5	-1843.8	-1541.8	-1795.3

Figures are modelled parameter estimates (95% confidence intervals).

BL1 = indicator for baseline 1; BL2 = indicator for baseline 2; BL3 = indicator for baseline 3; T6 = indicator for t = 6 months after start of intervention; T12 = indicator for t = 12 months after start of intervention; iv = indicator for intervention group; T6iv = interaction between T6 and iv; T12iv = interaction between T12 and iv.

#### a) Level of work-related burnout (MBI-HSS)

• Staff in IG had a significantly better score on *personal accomplishment *at 6 months, but there was no significant difference from staff in CG at 12 months.

• Staff in IG had a significantly better score on *emotional exhaustion *than staff in CG at both 6 months and 12 months.

• Staff in IG had a significantly better score on *depersonalisation *at 6 months, but there was no significant difference from staff in CG at 12 months.

#### b) Satisfaction with work (domains from SNCW items)

• *Professional role and development*: Staff in IG had a significantly better score than staff in CG at 12 months, though not at 6 months.

• *Work environment and staff collaboration: *Staff in IG had a higher score than staff in CG at 12 months and this difference was close to significance. There was no difference between IG and CG at 6 months.

• *Attitude towards residents' level of care: *Staff in IG had a higher score than staff in CG at 12 months and this difference was close to significance. There was no difference between IG and CG at 6 months.

• *Attitude towards individual contact with residents: *Staff in IG had a significantly better score than staff in CG at 6 months; at 12 months the score was again higher but the difference was not significant.

#### c) Self-rated health (SF12-v2)

• With respect to self-rated *physical health *there were no significant changes over time and no differences between IG and CG.

• With respect to self-rated *mental health*, staff in IG had a significant better score than CG at 6 months, but there was no significant difference from staff in CG at 12 months.

### Supplementary findings

Questionnaire responses from staff in the IG group showed that most were very positive about reminiscence; 90% considered it to be a good work tool, and many believed that it helped them to communicate with residents (68%). Most (76%) had used reminiscence in a more conscious manner than before, and 85% would recommend its implementation to other nursing homes.

Many of the IG staff interviewed considered that reminiscence had more of a short-term than a long-term effect. They gave many descriptions of residents who were otherwise withdrawn and quiet but during reminiscence sessions suddenly brightened up and began to talk, often surprising the nursing staff with their skills, knowledge and emotional expression.

Staff in each of the five IG nursing homes commented to the reminiscence trainers that they had become more aware of the contact they had with residents than before the study, and that they felt greater interest in eliciting residents' conversations and reactions. They felt that their use of reminiscence had resulted in a greater level of contact with the residents and more positive experiences. It had also become more natural for them to share personal aspects of their own lives with the residents, e.g. by using their own experiences as starting points for conversation; this had resulted in greater mutual understanding and more equality in their relationship.

## Discussion

The study results show only weak evidence for an effect of reminiscence on the nursing home residents. Reminiscence may have contributed to the observed delay in deterioration of cognitive function at 6 months in residents with moderate cognitive impairment at the start of the study, but no effect of reminiscence on cognitive function was observed after 12 months of intervention. Residents in the Intervention Group showed better scores on the quality of life subscale 'Response to surroundings' at 6 months, but again this difference was not apparent at 12 months. Reminiscence had no apparent effect on residents' agitated behaviour or general functioning.

The study findings show a more positive effect of reminiscence on the nursing home staff, although these effects were only small changes and not all reached statistical significance. Improvements were observed on all staff outcome measures except SF-12 self-rated physical health. At 12-months follow-up, staff in the Intervention Group had lower work-related burnout and greater satisfaction with work, including more positive attitudes towards the residents.

It is possible that reminiscence only has short-term effects (i.e. in the hours immediately after the session) that are not apparent in the assessment of residents' more general health status, functioning and well-being [39]. Other possible explanations of the relatively modest effects of reminiscence are discussed below.

### i) Other factors may have overshadowed the effect from reminiscence

The randomisation process attempted to adjust for differences between the two groups with respect to nursing home size, location and type. It was impossible, however, to adjust for possible differences in e.g. work culture, cooperation between staff groups, attitudes towards and expectations for residents. Despite a request to the nursing homes to avoid major organisational changes, these were sometimes unavoidable (e.g. new director, altered work routines) and for some months during the study there was negative national press coverage about the general quality of care in Danish nursing homes. Furthermore, the Danish public sector underwent a major structural reform in January 2007, although the nursing home managers judged its impact on the daily life of residents and staff to be limited.

### ii) The measurement instruments may have been inappropriate

Reminiscence may influence aspects of resident and staff well-being that were not captured by the instruments used. These instruments were selected on the basis of previous use in similar (Danish) contexts, evidence for acceptable psychometric properties and expectations that they would capture changes of interest. Assessment of resident cognition, behaviour and quality of life is not straightforward, however, and may be affected by background factors, e.g. educational level, physical health state, level of dementia [40,41]. In the current context the instruments showed high completion rates, good test-retest reliability and expected correlations between instruments (data not shown). Furthermore, the general deterioration observed over time in the residents' level of functioning has been seen in previous studies of nursing home residents and elderly with dementia e.g. with respect to ADRQL [42], GBS [27] and SIB-S [43]. Only a subset of the items in the Swedish SNCW instrument (Satisfaction with Nursing Care and Work Assessment) were used in the current study and these items were furthermore categorised into four dimensions using factor analysis; the results for this instrument are thus not directly comparable to those from other studies using the SNCW.

### iii) Aspects of the RCT design

Application of the RCT approach can be difficult for dynamic interventions where context is an important factor in determining whether and how an intervention will work, and where methodological issues arise related to size of target population, duration of follow-up and attrition [44]. Although the RCT design worked well overall there were some difficulties e.g. recruitment of nursing homes, uncertainty surrounding calculation of the necessary sample size (which instrument to use as a basis for calculation; sample included all residents, not only those with dementia), study participants were not blinded to randomisation group as is desirable in an RCT, and there were high drop-out rates for residents and staff - although this was expected from previous studies [42,45].

### iv) Reminiscence was not fully implemented in each site

Although the staff at each IG site were interested and willing to implement reminiscence, and used it to a large extent in every day care, it was the project group's general impression that the staff in many places lacked resources to make full use of the knowledge gained through the course.

The main reasons for a less than full implementation appeared to be:

• Lack of time to plan and use general and specific reminiscence

• Insufficient support from management, in that staff would have wanted more regular discussion of reminiscence activities at staff meetings, greater participation of management personnel in the training course, and more visible praise for those who were active with reminiscence

• Lack of interest in learning about and using reminiscence due to a lack of recognition of the importance of residents' social and emotional needs. There can be a belief that it is not possible for the nursing home to improve the individual resident's quality of life e.g. because of the necessary daily routines, general lack of privacy and insufficient staff numbers; in addition there is often a tendency to focus on physical well-being and treatment rather than psychological well-being and quality of life [46]. Changing the approach to nursing home care is a complex undertaking that often requires a shift from a routine task-oriented day to more holistic and flexible care centred on quality of life [47].

• Implementation in a research context: the reminiscence training was purposely similar for all five nursing homes in an attempt to make the intervention as similar as possible; it would otherwise have been natural to tailor the activities more specifically to the individual nursing home. Some 'golden opportunities' to exploit the potential in the reminiscence method were thus not followed up e.g. for some individual residents, refurnishing of communal areas, contact to local voluntary workers and agencies.

When the study began, not all residents in the IG nursing homes had their life histories documented, and where these were documented, there was uncertainty about how they could be used in routine care. It was the reminiscence trainer's hope that the relatives would be active contributors to improving the documentation of residents' life histories, but this was seldom the case. Only a few relatives began to consider how they could convey information about the resident's habits and interests to the nursing home staff, and these were typically those relatives who had previously discussed these issues with the staff. This did not appear to be due to a lack of interest in the resident's well-being, but more a lack of time and resources on the relatives' side. The reminiscence exhibitions were, however, very successful in drawing relatives to the nursing homes, and many relatives expressed surprise at the extent of reminiscence activities and their influence on the residents' behaviour.

While it would appear that reminiscence can be implemented into the daily life and routine of a nursing home, a full implementation of the method requires visible support from management, staff recognition of the importance of residents' social and emotional needs, time and energy to plan reminiscence activities, as well as varied types of reminiscence materials. Further studies would be useful to investigate whether particular groups of residents (e.g. with respect to cognitive level, social needs) or staff (e.g. age, ethnic background, prioritising of care duties) can gain most from reminiscence; how residents perceive reminiscence activities; whether other forms of reminiscence implementation can give more effective implementation; the nature of the relationship between the immediate short-lived effects of reminiscence sessions and any longer-term effects.

## Limitations of the study

The extent to which the participating nursing homes were representative of Danish nursing homes is unknown, although they did include examples of the different types of nursing home found in Denmark with respect to layout, size and general approach to nursing care. As expected, the drop-out rate among residents was high and mainly due to death. There was an element of self-selection bias due to the requirement of nursing homes/municipalities to be willing and able to participate in a research study with the extra burden of new learning and data collection and to provide self-financing. It is also possible that, despite the randomised matched approach, there were still variations between the two groups with respect to the daily environment and work culture.

Bias can have arisen from the nursing staff acting as proxy respondents for residents with respect to the assessment of the residents' functional level, agitated behaviour and quality of life (nursing staff also provided information on residents' sociodemographic and clinical details). The staff assessments may not have reflected the residents' true functional level - there is evidence that proxy responses are not always consistent with patient assessments, especially in relation to more subjective aspects such as emotional functioning and quality of life [48-50]. Furthermore, a resident could be assessed by different staff throughout the study. This bias is not considered to be of high significance, however, as 87% and 75% of the staff at the 6-month and 12-month follow-ups, respectively, had assessed the resident at baseline; furthermore, 70% and 95% of the staff at baseline and 12-months follow-up, respectively, had been primary carer for the resident for at least 6 months prior to making the assessment.

The reminiscence approach used here was broader than the typical, in that all staff were taught in all three forms (spontaneous, specific and general); all residents (both with and without dementia) were included in the reminiscence activities and the residents' relatives were strongly encouraged to actively participate. The training course was also atypical in that five nursing homes were taught simultaneously (rather than one at a time), and there were more frequent follow-up visits by the reminiscence trainers, e.g. to deliver reminiscence boxes and explain their content and potential use.

## Conclusions

The results from this randomised intervention study indicate that the use of reminiscence has little long-term effect on residents of nursing homes. However, the nursing staff in the Intervention Group experienced greater satisfaction with professional roles and developed a more positive view of the residents and of their work environment. It would seem that reminiscence activities can help staff to view residents in a more personal light, with greater recognition of previous life experiences, and thus offer opportunities for more regular engagement in mutually enjoyable social activities.

## Competing interests

All authors declare that the answer to the questions on the competing interest form are all No and therefore have nothing to declare.

## Authors' contributions

As the primary reminiscence trainer, MK developed the reminiscence course and material and ran the training courses. AMJ was responsible for recruitment of nursing homes and implementation of reminiscence, in particular the use of reminiscence boxes, posters and exhibitions. CG and CH contributed to study design and were responsible for data collection, analysis and reporting. JS contributed to study design, data analysis and reporting. All authors read and approved the final manuscript.

## Pre-publication history

The pre-publication history for this paper can be accessed here:

http://www.biomedcentral.com/1471-2318/10/33/prepub
